# Anti-Inflammatory Effects of Huangqin Decoction on Dextran Sulfate Sodium-Induced Ulcerative Colitis in Mice Through Regulation of the Gut Microbiota and Suppression of the Ras-PI3K-Akt-HIF-1α and NF-κB Pathways

**DOI:** 10.3389/fphar.2019.01552

**Published:** 2020-01-20

**Authors:** Min-yao Li, Hui-juan Luo, Xue Wu, Yu-hong Liu, Yu-xuan Gan, Nan Xu, Yao-min Zhang, Shu-hua Zhang, Chang-lin Zhou, Zi-ren Su, Xiao-qi Huang, Xue-bao Zheng

**Affiliations:** ^1^ Mathematical Engineering Academy of Chinese Medicine, Guangzhou University of Chinese Medicine, Guangzhou, China; ^2^ Dongguan Songshan Lake Yidao TCM Clinic, Dongguan, China; ^3^ Graduate School, Guangdong Medical University, Dongguan, China

**Keywords:** ulcerative colitis, Huangqin decoction, gut microbiota, Ras-PI3K-Akt-HIF-1α pathway, NF-κB pathway

## Abstract

**Objective:**

Huangqin decoction (HQD), a classical traditional Chinese medicinal formula, has been commonly used to treat gastrointestinal diseases for thousands of years. We investigated the anti-inflammatory effects and underlying mechanisms of HQD on dextran sulfate sodium (DSS)-induced ulcerative colitis (UC).

**Methods:**

Experimental mice were given 3% DSS, and HQD (2.275, 4.55, and 9.1 g/kg), or mesalazine (ME, 200 mg/kg) orally for 7 days. Body weight loss, disease activity index (DAI), colon length, histology, and levels of inflammatory cytokines were measured to evaluate the effects of HQD on colitis. The effects of HQD on the Ras-phosphoinositide-3-kinase (PI3K)-Akt-hypoxia inducible factor 1 alpha (HIF-1α) and nuclear factor-kappa B (NF-κB) pathways were evaluated by Western blot analysis. In addition, the gut microbiota was characterized using high-throughput Illumina MiSeq sequencing.

**Results:**

The results showed that HQD significantly reduced the body weight loss, ameliorated DAI, restored colon length, and improved the intestinal epithelial cell barrier in mice with DSS-induced colitis. The messenger RNA (mRNA) expression levels of inflammatory mediators were decreased following HQD treatment. Furthermore, the Ras-PI3K-Akt-HIF-1α and NF-κB pathways were significantly inhibited by HQD. Finally, treatment with HQD resulted in recovery of gut microbiota diversity.

**Conclusions:**

HQD ameliorates DSS-induced colitis through regulation of the gut microbiota, and suppression of Ras-PI3K-Akt-HIF-1α and NF-κB pathways. Our results suggested that HQD may be a potential candidate for treatment of UC.

**Graphical Abstract:**
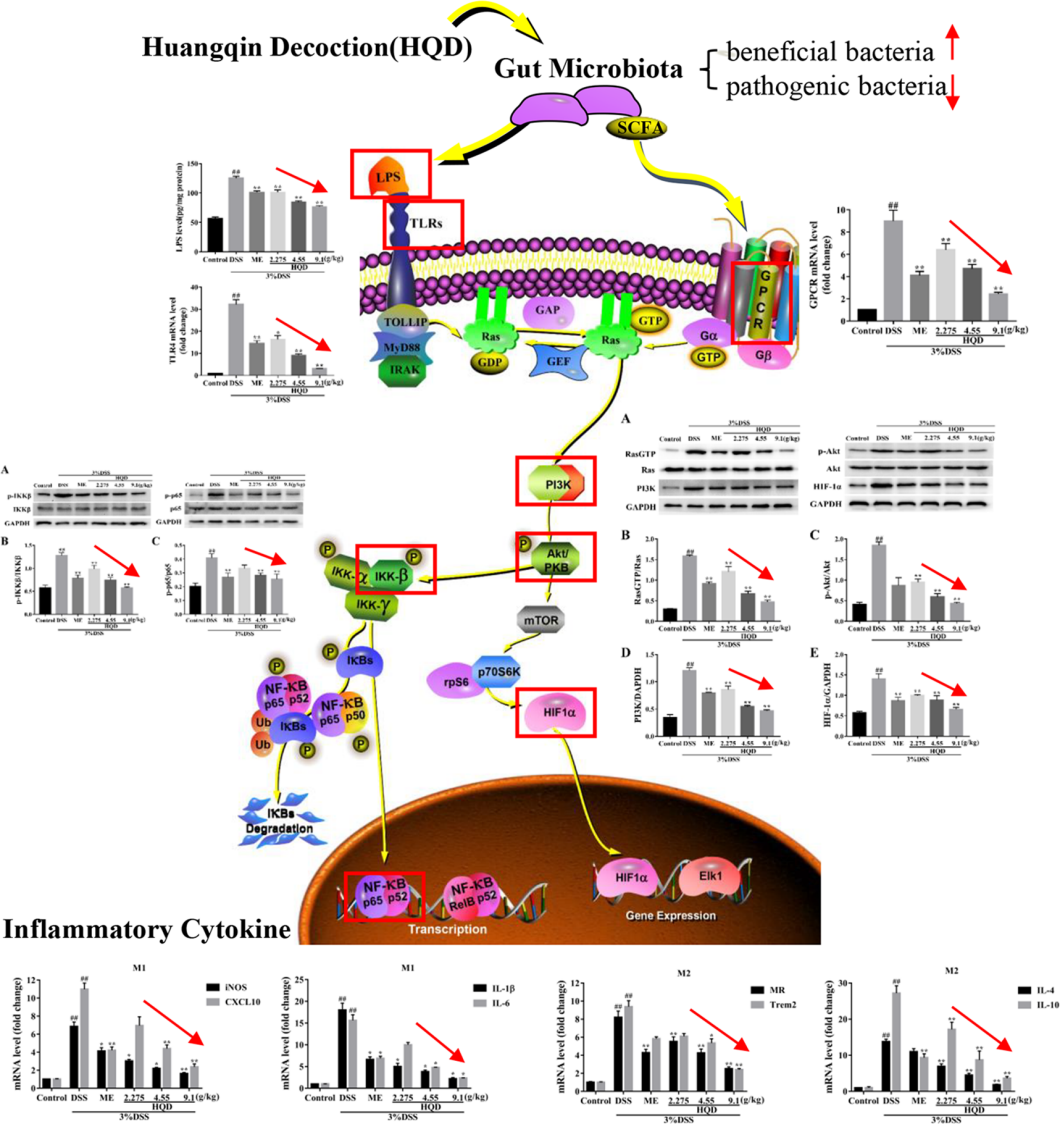
This study demonstrated that supplementation of Huangqin decoction (HQD) attenuated dextran sulfate sodium (DSS)-induced ulcerative colitis by regulating the gut microbiota and suppressing Ras-PI3K-Akt-HIF-1α and NF-κB pathways in mice.

## Introduction

Ulcerative colitis (UC) is a chronic inflammatory disease of the colon and rectum with unclear etiology. UC and Crohn's disease (CD) are collectively referred to as inflammatory bowel disease (IBD) (Kaser et al., 2009). The main clinical symptoms of UC are diarrhea, bloody stools, abdominal pain, tenesmus, and weight loss due to malabsorption (Kornbluth et al., 2010). Recent studies showed that UC is a chronically recurring inflammatory disorder, characterized by intestinal inflammation and dysregulation of the mucosal epithelial barrier, which is one of the three greatest risk factors for development of colorectal cancer (CRC). Epidemiological studies have shown that CRC (UC-CRC) is a serious UC-related complication, resulting in one-sixth IBD-related deaths (Munkholm, 2003). Current treatments for UC include mesalazine (ME), sulfasalazine, immunosuppressive drugs, corticosteroids, and interferons. However, these drugs are associated with side effects such as nausea, headache, and dizziness (Ross and Cohen, 2004). In contrast, many complementary and alternative medicinal (CAM) approaches have been shown to be effective for treatment of IBD (Maciej et al., 2014). Development of novel efficient and safe therapeutic strategies for treatment of IBD is needed.

Huangqin decoction (HQD, Scutellariae Radix decoction), a traditional herbal formula from *Shang Han Lun*, has been used for nearly 1,800 years. Use of this formula is indicated as follows: “When the disease occurs both in Taiyang and Shaoyang, characterized by diarrheas, Huangqin Tang is prescribed.” The evil heat from Taiyang and Shaoyang forces Yangming, resulting in gastrointestinal dysfunction. Clinical studies have shown that HQD is safe and effective for treatment of complex gastrointestinal symptoms (Liu and Cheng, 2012). The classical formula consists of four components: the roots of *Scutellaria baicalensis* Georgi (skullcap, 9 g), *Glycyrrhiza uralensis* Fisch. (licorice, 6 g), *Paeonia lactiflora* Pall. (peony, 6 g), and the fruit of *Ziziphus jujuba* Mill. (jujube, 49 g). Recent studies showed that HQD alleviated inflammation through suppression of the nuclear factor-kappa B (NF-κB) pathway and regulation of the gut microbiota to improve the intestinal microenvironment in mouse models of dextran sulfate sodium (DSS)-induced UC (Chen et al., 2016; Zou et al., 2016; Yang et al., 2017). However, these studies did not clarify how the gut microbiota exerted effects on inflammatory pathways. We hypothesized that HQD influenced activation of the Ras-PI3K-Akt-HIF-1α pathway, resulting in inhibition of the NF-κB pathway and improvement in the gut microbiota.

We investigated the molecular mechanisms of HQD in a murine model of DSS-induced colitis by measuring the expression levels of inflammatory cytokines, receptors, and proteins in the Ras-PI3K-Akt-HIF-1α and NF-κB pathways. Furthermore, we profiled the gut microbiota using Illumina HiSeq 2500 sequencing of the bacterial 16S ribosomal DNA (rDNA) gene V3–V4 region.

## Materials and Methods

### Drugs and Reagents

The four medicinal herbs contained in HQD (skullcap, licorice, peony, and jujube) were purchased from The First Affiliated Hospital of Guangzhou University of Traditional Chinese Medicine (Guangzhou, China). All herbal materials were accredited by Professor Jiannan Chen of Guangzhou University of Chinese Medicine, where four voucher specimens (voucher 18-09-22, 18-09-23, 18-09-24, 18-09-25) were deposited. The standards paeoniflorin, liquiritin, baicalin, oroxylin A-7-glucuronide, wogonoside, baicalein, wogonin, and oroxylin A were purchased from Dalian Meilun Biotechnology Co., Ltd. (Dalian, China). DSS was obtained from MP Biomedicals, LLC, France. ME was purchased from Losan Pharma GmbH, Germany. Primary antibodies [NF-κB p65, p-p65, inhibitor kappa B kinase β (IKKβ), phosphorylated IKKβ (p-IKKβ), phosphoinositide-3-kinase (PI3K), Akt, p-Akt, Ras, RasGTP, hypoxia inducible factor 1 alpha (HIF-1α), and glyceraldehyde 3-phosphate dehydrogenase (GAPDH)] and secondary antibodies were purchased from Affinity Biosciences (OH, USA). Enzyme-linked immunosorbent assay (ELISA) kits for lipopolysaccharide (LPS) and myeloperoxidase (MPO) were purchased from Shanghai Enzyme-linked Biotechnology Co., Ltd. (Shanghai, China). Primers for G protein-coupled receptors (GPCR), toll-like receptor 4 (TLR4), mannose receptor (MR), triggering receptor expressed on myeloid cells 2 (Trem2), inducible nitric oxide synthase (iNOS), CXC chemokine ligand 10 (CXCL10), interleukin-1β (IL-1β), interleukin-6 (IL-6), interleukin-4 (IL-4), interleukin-10 (IL-10), and GAPDH were synthesized by Shanghai Sangon Biotech Co., Ltd. (Shanghai, China). All reagents and chemicals were of analytical grade.

### Composition and Preparation of Huangqin Decoction

HQD was prepared by grinding 90 g of skullcap, 60 g of licorice, 60 g of peony, and 490 g of jujube, then boiling at 100°C for 30 min in a 10X volume of distilled water. After filtration, the residue was extracted using an 8X volume of distilled water. The filtrates were combined in a container and stored at 4°C for subsequent animal experiments.

The HQD extract was analyzed using a Shimadzu LC-20AT Prominence high-performance liquid chromatography (HPLC) system equipped with a diode array detector. Chromatographic separation was performed on Diamonsil C18 (250 mm × 4.6 mm, 5 μm) maintained at 30°C. The mobile phase flow rate was 1 ml/min. The mobile phases were 0.1% (v/v) formic acid (A) and acetonitrile (B). The gradient elution program was as follows: 0–15 min, 95–95% A (v/v), 5–5% B (v/v); 15–30 min, 95–85% A, 5–15% B; 30–60 min, 85–77% A, 15–23% B; 60–90 min, 77–55% A, 23–45% B; 90–110 min, 55–40% A, 45–60% B; 110–115 min, 40–90% A, 60–5% B; 115–120 min, 40–95% A, 60–5% B. The injection volume was 10 μl and the detection wavelength was 280 nm (Yang et al., 2017).

### Experimental Animals and Grouping

Male BALB/c mice (22–26 g, aged 6–8 weeks) were purchased from the Experimental Animal Center in Guangzhou University of Chinese Medicine (Guangzhou, China). All experimental mice were housed in cages under standard environmental conditions (18–25°C, 60–70% humidity, 12 h light and dark cycle) with free access to food and sterile water. All animal study protocols were performed in accordance with the approved guidelines of the Animal Experimentation Ethics Committee at Guangzhou University of Chinese Medicine.

The animals were allowed to acclimate for 7 days, then were divided into six groups (*n =* 12): control, DSS, DSS with ME (ME, 200 mg/kg), and DSS with HQD (HQD, 2.275, 4.55, and 9.1 g/kg). The control group received normal sterile water without DSS. The other mice were allowed to drink 3% DSS freely for 7 days (Stefan et al., 2007). The volumes of distilled water or DSS solution consumed by all of the animals were measured daily, and there were no significant differences in water consumption among the groups. HQD and ME were administered daily by oral gavage (Chen et al., 2016; Wang et al., 2018).

### Evaluation of Colitis

Body weight, stool consistency, and rectal bleeding were monitored daily by an observer blinded to the study details. Disease activity index (DAI) was calculated as follows: DAI = (weight loss score + stool characters score + hematochezia score)/3 (Murano et al., 2000). The mice were sacrificed by cervical dislocation under anesthesia, the colorectum were removed, and the lengths were recorded. Portions of the distal colorectum were fixed in 10% formalin and embedded in paraffin. Then they were stained with hematoxylin and eosin (H&E) and periodic acid-Schiff (PAS) according to standard protocols. Histological scoring of H&E staining was performed in a blinded manner as described previously (Murano et al., 2000). Tissue expression of neutral mucins in goblet cells was determined as a function of PAS staining.

### Enzyme-Linked Immunosorbent Assay

The colorectal tissues from each group were cut into pieces and homogenized in phosphate-buffered saline (PBS) buffer (pH = 7.4) to extract total protein. The precipitates were collected following centrifugation at 3,000 rpm for 20 min at 4°C. The levels of LPS and MPO were determined using ELISA kits according to the manufacturer's protocols. The amount of total protein in each sample was measured using the bicinchoninic acid assay (BCA) protein assay kit (Pierce, USA).

### Quantitative Real-Time Polymerase Chain Reaction Analysis

Total RNA was extracted from the colon using TRIzol reagent (Invitrogen Life Technologies, MA, USA) according to the manufacturer's protocols. The extracted RNA was reverse transcribed to complementary DNA (cDNA) using a reverse transcriptase kit (Thermo Scientific, MA, USA). The cDNA was analyzed by quantitative real-time PCR (qRT-PCR) using ChamQ SYBR qPCR Master Mix (Vazyme Biotech Co., Ltd., Nanjing, China) and CFX Manager software (Bio-Rad Laboratories Inc.). Each reaction was subjected to the following cycling conditions: a pre-cycling stage at 95°C for 10 min, followed by 40 cycles at 95°C for 15 s, and 60°C for 20 s. The 2^−ΔΔCt^ method was used to determine the messenger RNA (mRNA) expression levels of cytokines and receptors in colon tissue relative to the expression of GAPDH. The expression levels of M1 macrophage markers (iNOS, CXCL10), M2 macrophage markers (MR, Trem2), pro-inflammatory cytokines (IL-1β, IL-6), anti-inflammatory cytokines (IL-4, IL-10), and receptors (TLR4, GPCR) were evaluated. The sequences of the primers used in this study are listed in **Table 1**.

**Table 1:** Sequences of primers used for quantitative real-time PCR (qRT-PCR).

Target gene	Nucleotide sequence of primer (5' to 3')	Reverse
	Forward	
*iNOS*	GATGTGCTGCCTCTGGTCTTGC	CAGCCACATTGATCTCCGTGACAG
*CXCL10*	ATGAACCCAAGTGCTGCCG	TTCATCGTGGCAATGATCTCAACA
*MR*	AAGCCTGACACCGCCGAGAG	CTGAAGCCGCACGAACTGAAGG
*Trem2*	ACTTATGACGCCTTGAAGCACTGG	CCTCGGAGACTCTGACACTGGTAG
*IL-1β*	GCACTACAGGCTCCGAGATGAAC	AGGCTTGTGCTCTGCTTGTGAG
*IL-6*	TGAACAACGATGATGCACTTGCAG	TAGCCACTCCTTCTGTGACTCCAG
*IL-4*	GGTCTCAACCCCCAGCTAGT	GCCGATGATCTCTCTCAAGTGAT
*IL-10*	AGCTGGACAACATACTGCTAACCG	CTTCACCTGCTCCACTGCCTTG
*TLR4*	ACAAGGCATGGCATGGCTTACAC	TGTCTCCACAGCCACCAGATTCTC
*GPCR*	GCCTCATCGTCATCGCCAACC	GGAAGAAGCAGCCAGCAGGTG
*GAPDH*	AATGGTGAAGGTCGGTGTGAACG	TCGCTCCTGGAAGATGGTGATGG

### Western-Blot Analysis

Colorectal tissues were homogenized in radioimmunoprecipitation assay (RIPA) buffer containing phenylmethylsulfonyl fluoride (PMSF) and protease inhibitor. Total protein was extracted using a protein extraction kit following the manufacturer's instructions. Total protein was quantified, separated by 10% sodium dodecyl sulfate polyacrylamide gel electrophoresis (SDS-PAGE), and transferred to polyvinylidene fluoride (PVDF) membranes. The membranes were blocked in 5% nonfat dried milk and incubated with primary antibodies (NF-κB p65, p-p65, IKKβ, p-IKKβ, PI3K, Akt, p-Akt, Ras, RasGTP, HIF-1α, and GAPDH, all at 1:1,000 dilutions) overnight at 4°C. The membranes were then incubated with secondary antibodies for 90 min at room temperature. Protein bands were quantified using ImageJ software with GAPDH as the internal control.

### Fecal Deoxyribonucleic Acid Extraction and Illumina Hiseq 2500 Sequencing

Mice feces in the colon were collected after sacrifice. Fecal genomic DNA was extracted using HiPure soil DNA kits (or HiPure stool DNA kits) (Magen, Guangzhou, China) according to the manufacturer's instructions. The 16S rDNA V3–V4 region of the ribosomal RNA (rRNA) was amplified using the forward primer 341F (5'-CCTACGGGNGGCWGCAG-3') and the reverse primer 806R (5'-GGACTACHVGGGTATCTAAT-3') by PCR. Amplicons were purified using the AxyPrep DNA gel extraction kit (Axygen Biosciences, Union City, CA, U.S.) according to the manufacturer's instructions and quantified using an ABI StepOnePlus real-time PCR system (Life Technologies, Foster City, USA). Purified amplicons were paired-end sequenced (2 × 250) using an Illumina platform.

### Bioinformatics Data Analysis

Noisy sequences of raw tags were filtered using QIIME (version 1.9.1). The effective tags were clustered into operational taxonomic units (OTUs) of ≥97% similarity using UPARSE pipeline. The taxonomy of each sequence was classified into organisms using RDP classifier (version 2.2) based on the SILVA database with confidence threshold values ranging from 0.8 to 1. Alpha diversity analysis was used to compare the diversity between groups. The dominant bacterial community difference between groups were screened using Metastats (version 20090414) and LEfSe software (version 1.0).

### Statistical Analysis

All experimental data were presented as the mean ± the standard error of the mean (SEM) using Statistical Product and Service Solutions (SPSS) software (version 23.0). Statistical analyses consisted of one-way ANOVA (for parametric data) followed by the least significant difference (LSD) test and the Mann-Whitney U test (for non-normal data). Value of *p* < 0.05 was considered statistically significant.

## Results

### Components of Huangqin Decoction

**Figure 1:**
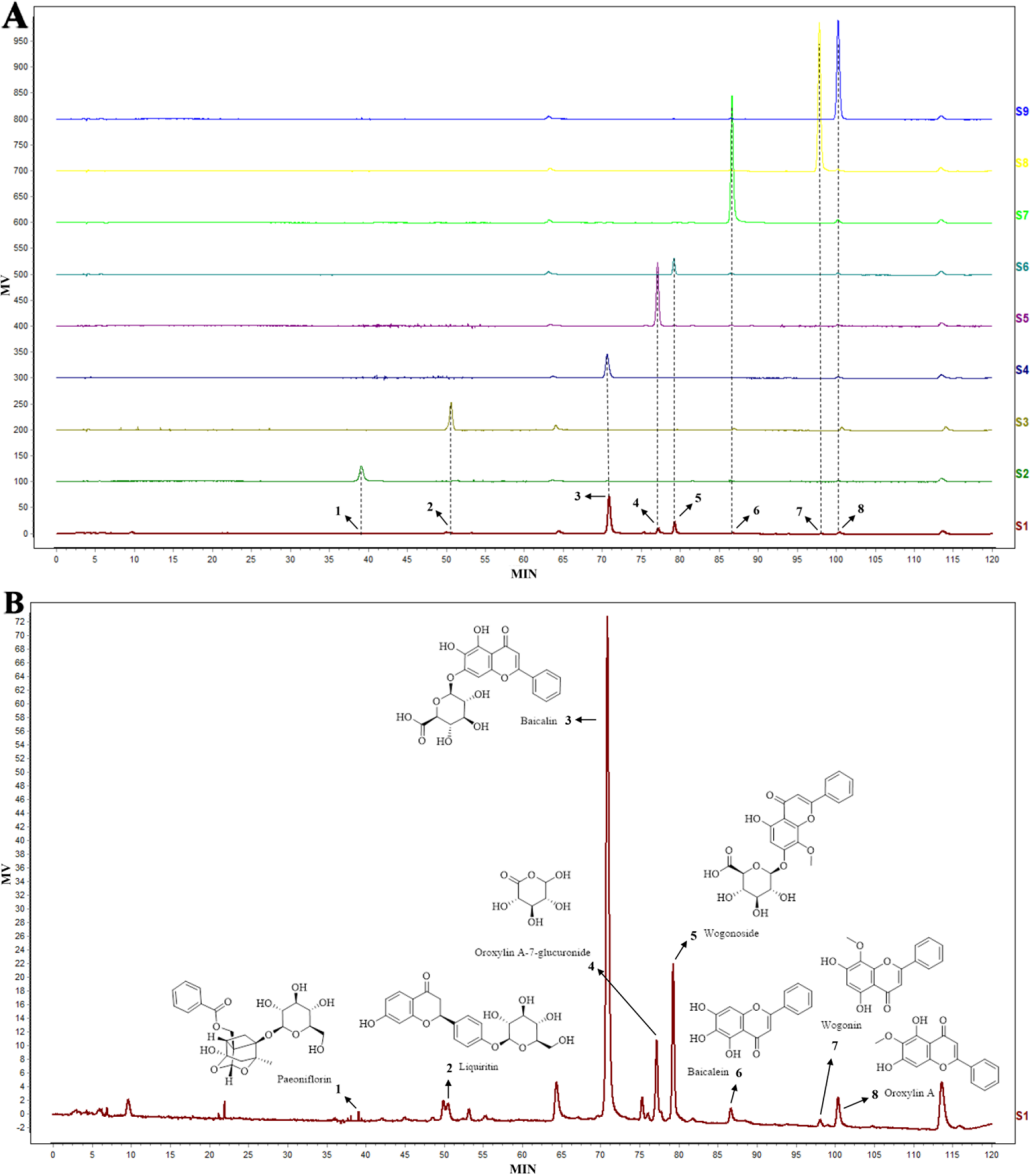
The high-performance liquid chromatography (HPLC) chromatogram **(A)** of Huangqin decoction (HQD) extract (S1) and standards (S2–9), and the structural formula of chemical compounds in HQD **(B)**. The eight components in HQD were paeoniflorin (1), liquiritin (2), baicalin (3), oroxylin A-7-glucuronide (4), wogonoside (5), baicalein (6), wogonin (7), and oroxylin A (8), respectively.

The components of HQD were analyzed by HPLC and compared with standards. As shown in **Figure 1**, there were eight components in HQD, including paeoniflorin, liquiritin, baicalin, oroxylin A-7-glucuronide, wogonoside, baicalein, wogonin, and oroxylin A. Among these components, baicalin and wogonoside were identified as the two major components. Baicalin and wogonoside have been previously shown to induce anti-inflammatory effects against DSS-induced colitis in mice (Sun et al., 2015; Zhang et al., 2017).

### Huangqin Decoction Reduced Inflammatory Indices in Dextran Sulfate Sodium-Induced Colitis

**Figure 2:**
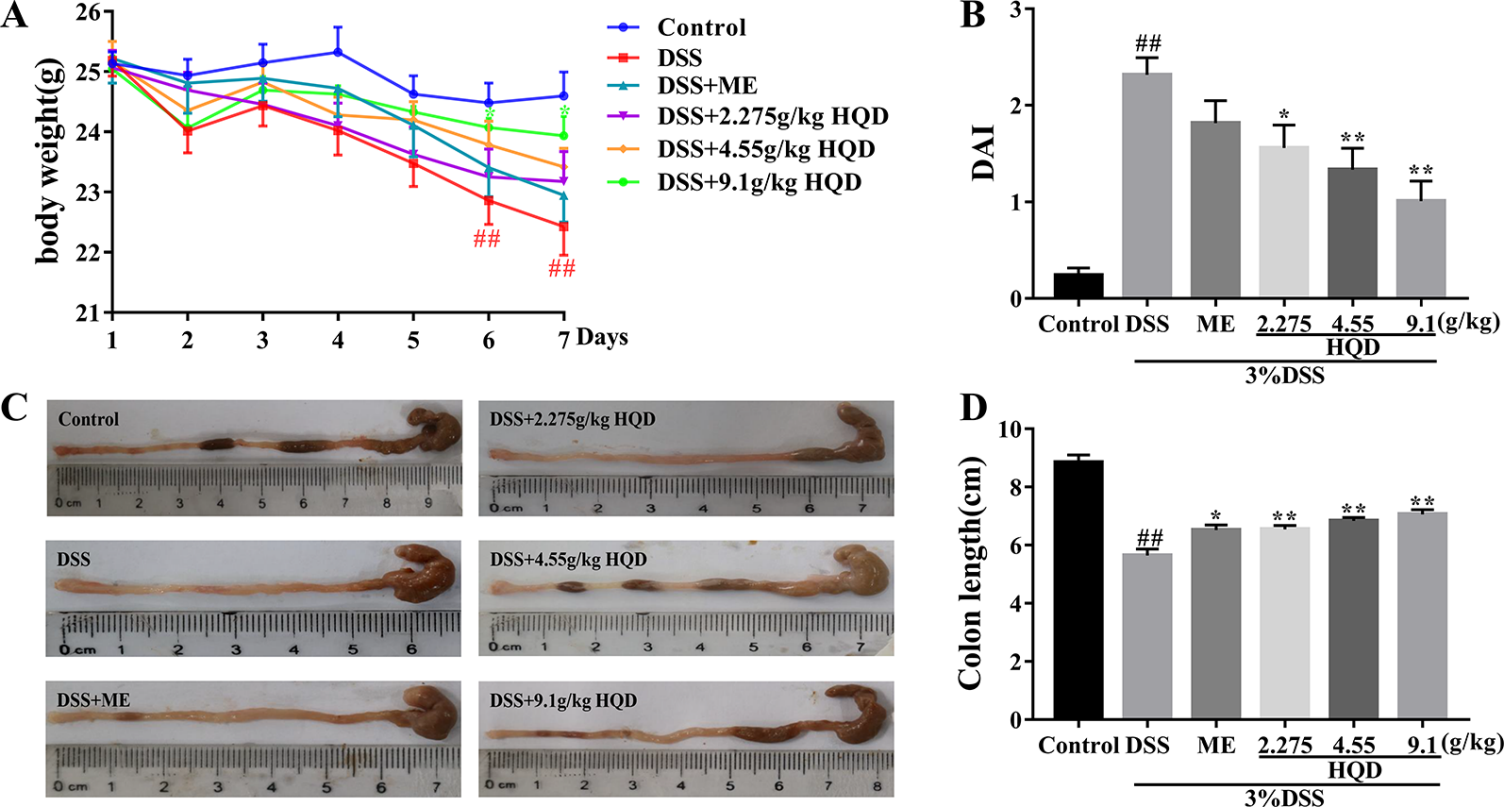
Huangqin decoction (HQD) ameliorated inflammation in dextran sulfate sodium (DSS)-induced acute colitis. **(A)** The daily body weight changes from first day to seventh day. **(B)** The disease activity index (DAI) in mice. **(C)** Representative images of colon lengths from a macroscopic perspective. **(D)** Quantitative measurement of colon length. Data are presented as the means ± SEM (*n =* 12). ^##^ *P* < 0.01 *vs*. control group; *P < 0.05, **P < 0.01 *vs*. DSS group.

HQD was given to mice treated with 3% DSS for 7 days (5–7 ml of water consumption per mouse, **Supplementary Table 1**) to evaluate the anti-inflammatory effects of HQD against UC. As shown in **Figure 2**, there were no significant differences among the groups in body weight change on the first day of the experiment. On the day 7, mice treated with 3% DSS alone showed significant weight loss compared to control group (*p* < 0.01), and treatment with 9.1 g/kg of HQD alleviated DSS-induced weight loss (*p* < 0.05). Furthermore, mice treated with 2.275 and 4.55 g/kg of HQD or ME lost less weight than the mice in the DSS group.

The DAI was used to evaluate the severity of colitis symptoms as previously described (Murano et al., 2000). As shown in **Figure 2**, the DAI score of the DSS group was significantly higher than that in the control group (*p* < 0.01). Treatment with HQD reduced DAI scores even lower than ME group, especially significant changes were observed in 4.55 g/kg of HQD (*p* < 0.05) and 9.1 g/kg of HQD (*p* < 0.01) groups compared to the DSS group. In addition, previous researches indicated that the DAI score was inversely associated with the length of colon (Feifei et al., 2015). The colon length in DSS group was much shorter than that of control group (*p* < 0.01) in **Figures 2C, D**. In contrast, the colon length in HQD groups as well as ME group increased dramatically compared with DSS group (all *p* < 0.01).

### Huangqin Decoction Improved the Intestinal Epithelial Cell Barrier in Dextran Sulfate Sodium-Induced Colitis

**Figure 3:**
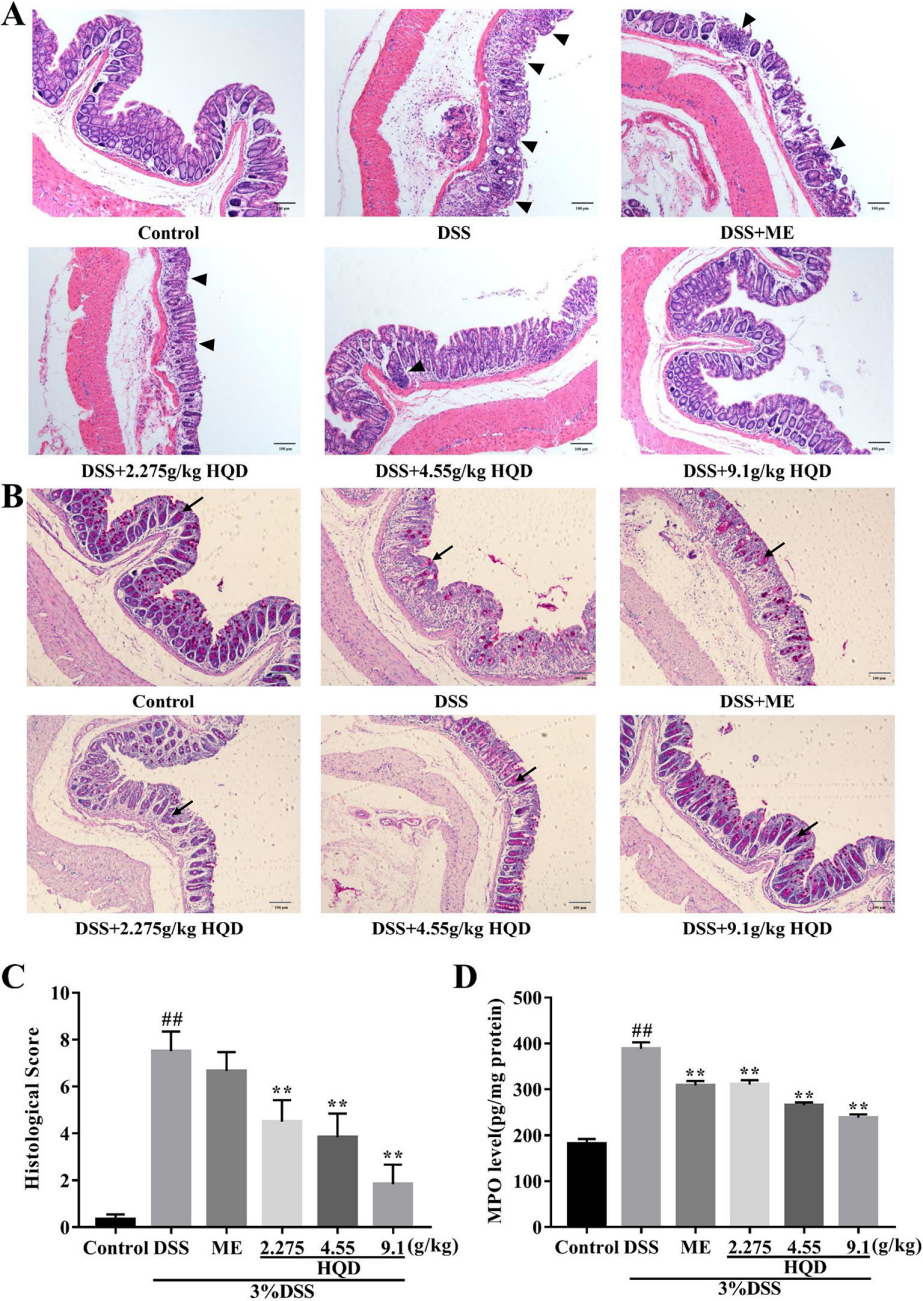
Effects of Huangqin decoction (HQD) on the intestinal epithelial cell barrier in dextran sulfate sodium (DSS)-induced acute colitis. **(A)** H&E staining of the formalin-fixed sections of colon. Arrows indicates the infiltration of intestinal epithelial cells. **(B)** Periodic acid-Schiff (PAS) staining of goblet cells. Arrows indicates the neutral mucins of goblet cells. **(C)** Histological scores and **(D)** myeloperoxidase (MPO) activity of colon tissue. Data are presented as the means ± SEM (*n =* 6–10). ^##^ *p* < 0.01 *vs*. control group; **p < 0.01 *vs*. DSS group.

The colonic epithelium consists of a variety of epithelial cells, such as enterocytes, goblet cells, Paneth cells, and enteroendocrine cells (Nick, 2014; Odenwald and Turner, 2016). Intestinal goblet cells secrete mucins to create a first layer of defense against invasion of foreign pathogens (Kim and Ho, 2010). H&E staining (**Figure 3**) showed that the DSS group exhibited inflammatory cell infiltration, disruption of crypt structure, and loss of enterocytes in the mucosa, which suggested severe intestinal barrier damage. Treatment with 9.1 g/kg of HQD alleviated DSS-induced gut epithelial morphological changes (*p* < 0.01). Furthermore, DSS induced severe depletion of goblet cells (visualized using PAS staining) (**Figure 3**). Depletion of goblet cells by DSS was reversed by HQD administration.

MPO activity, a marker of neutrophil infiltration, has been used to assess inflammation in colon tissue (Sittisart et al., 2016). As shown in **Figure 3**, MPO activity was markedly increased in DSS-treated mice compared to that in the control group (*p* < 0.01), which suggested that DSS induced neutrophil recruitment. In contrast, treatment with HQD or ME resulted in decreased MPO activity compared with the DSS group (all *p* < 0.01). Our results showed that HQD significantly prevented infiltration to protect the intestinal epithelial cell barrier.

### Huangqin Decoction Suppressed the Ras Signaling Pathway Through Regulation of TLR4 and GPCR

**Figure 4:**
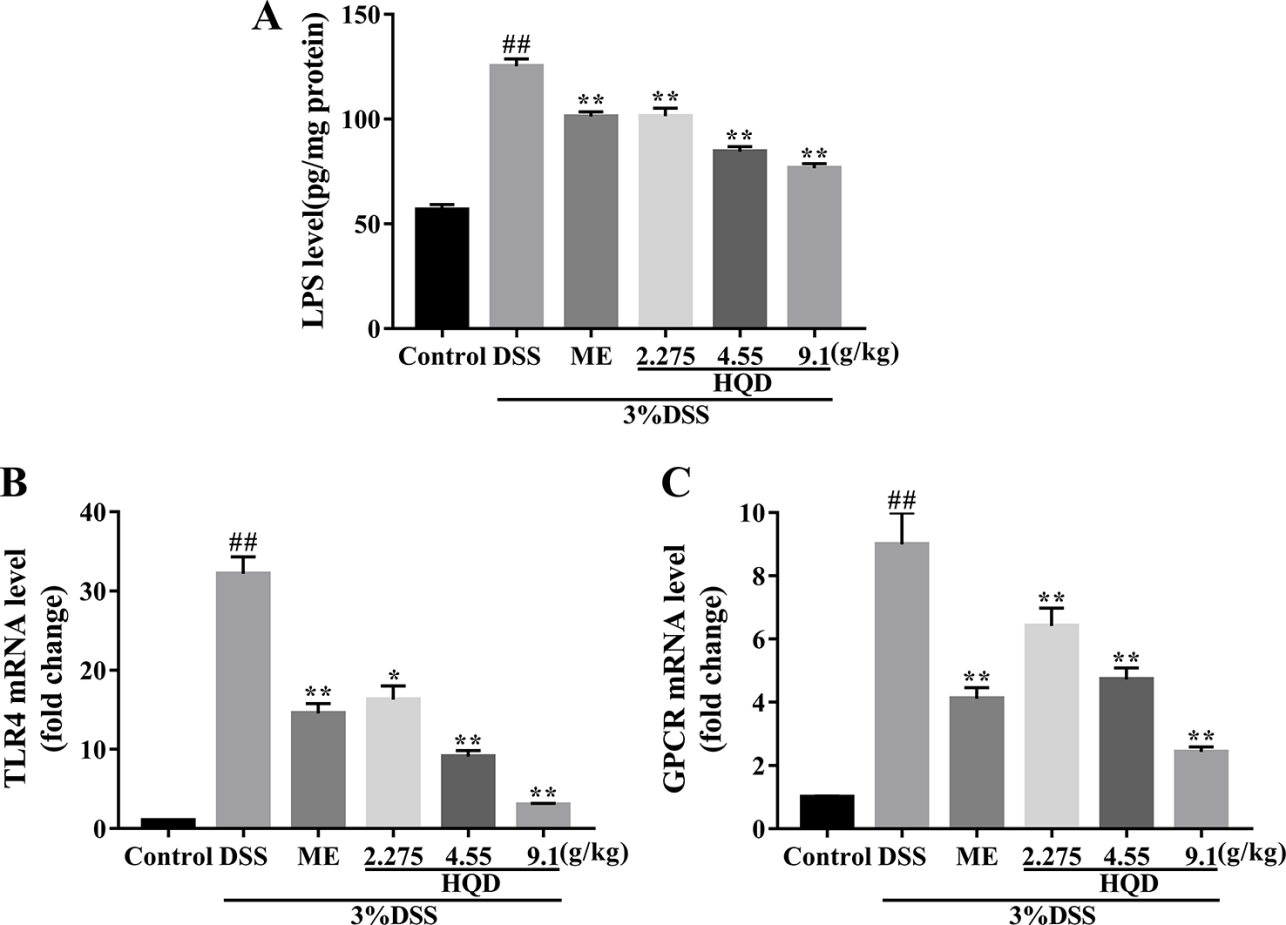
Effects of Huangqin decoction (HQD) on colonic level of lipopolysaccharide (LPS) **(A)** as determined by ELISA and the colonic messenger RNA (mRNA) levels of TLR4 **(B)** and G protein-coupled receptors (GPCR) **(C)** in dextran sulfate sodium (DSS)-induced colitis mice. Data are presented as the means ± SEM (*n* = 4–8). ^#^ *p* < 0.05, ^##^ *p* < 0.01 *vs*. control group; **p < 0.01 *vs*. DSS group.

LPS activates the Ras signaling pathway by binding to TLR4 (Ng et al., 2017), resulting in increased production of several inflammatory mediators and effector molecules in macrophages (Kim and Kim, 2005). We showed that colonic LPS levels in the DSS group were significantly higher than those in the control group (*p* < 0.01, **Figure 4**). Treatment with HQD reversed DSS-induced increases in LPS (all *p* < 0.01). In addition, DSS treatment resulted in increased expression of TLR4 mRNA compared with the control group (*p* < 0.01, **Figure 4**). Treatment with HQD significantly reduced DSS-induced expression of TLR4 mRNA, resulting in decreased activation of the TLR4 pathway.

GPCR control a variety of primary intracellular signals, and can be constitutively active in the absence of ligand binding (Bais et al., 1998). Previous studies showed that GPCR activated the Ras and PI3K/Akt pathways, or their downstream targets (Montaner et al., 2001). We evaluated the mRNA expression of GPCR. Treatment with DSS resulted in significantly increased GPCR activation (*P* < 0.01, **Figure 4**), which suggested a strong inflammatory response. Treatment with HQD or ME reduced the expression of GPCR mRNA in a dose-dependent manner (*P* < 0.01).

### Huangqin Decoction Regulated the Levels of Macrophage Markers and Inflammatory Cytokines

**Figure 5:**
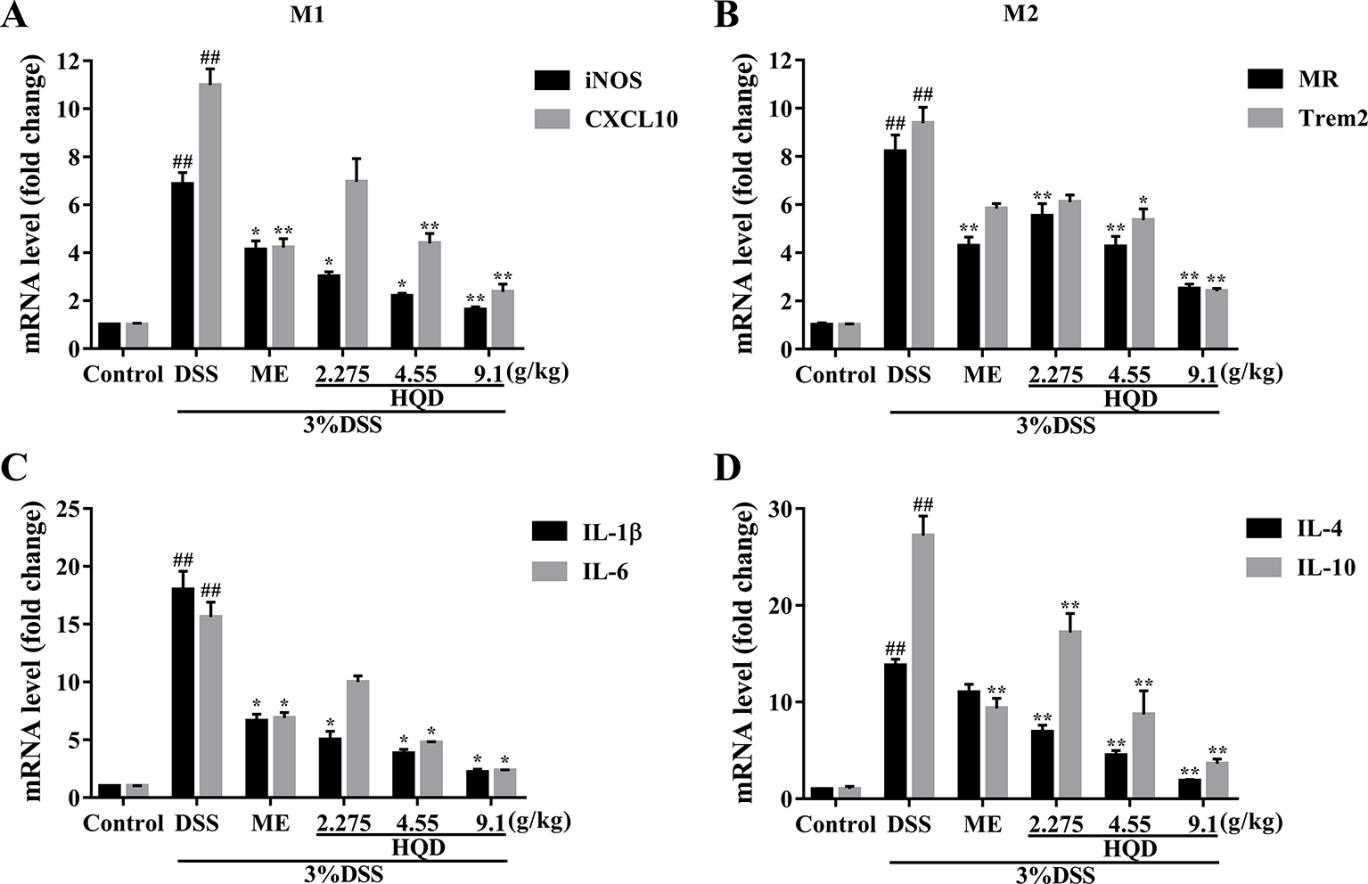
Effects of Huangqin decoction (HQD) on the colonic messenger RNA (mRNA) levels of macrophage markers and inflammatory cytokines as determined by quantitative real-time PCR (qRT-PCR) in dextran sulfate sodium (DSS)-induced colitis mice. The expression levels of iNOS and CXCL10 **(A)**, MR and Trem2 **(B)**, IL-1β and IL-6 **(C)**, IL-4 and IL-10 **(D)**. ^##^ *p* < 0.01 *vs*. control group; *p < 0.05, **p < 0.01 *vs*. DSS group.

Macrophages can be categorized into M1 phenotype and M2 phenotype macrophages (Ruffell et al., 2012). M1 macrophages produce high levels of proinflammatory cytokines, and M2 macrophages exert anti-inflammatory effects (Ning-Bo et al., 2014). As shown in **Figure 5**, the levels of inflammatory mediators, including macrophage markers and inflammatory cytokines, were higher in the DSS group than those in the control group (all *p* < 0.01). Treatment with HQD significantly lowered the expression of iNOS and CXCL10, which are major M1 markers, compared to the DSS group (*p* < 0.01 and *p* < 0.05, respectively, **Figure 5**). The levels of pro-inflammatory cytokines (IL-1β and IL-6) involved in M1 macrophage polarization were significantly reduced in response to treatment with 9.1 g/kg of HQD (*p* < 0.01 and *p* < 0.05, **Figure 5**). Treatment with HQD also downregulated the expression of M2 macrophage makers such as MR and Trem2 compared to the DSS group (**Figure 5**). These markers have been shown to exert anti-inflammatory effects (Luzina et al., 2012). Lower expression of the anti-inflammatory cytokines IL-4 and IL-10 were observed in HQD-treated mice (all *p* < 0.01, **Figure 5**).

### Huangqin Decoction Attenuated Inflammation Through Inhibition of the Ras-PI3K-Akt-HIF-1α and NF-κb Pathways

**Figure 6:**
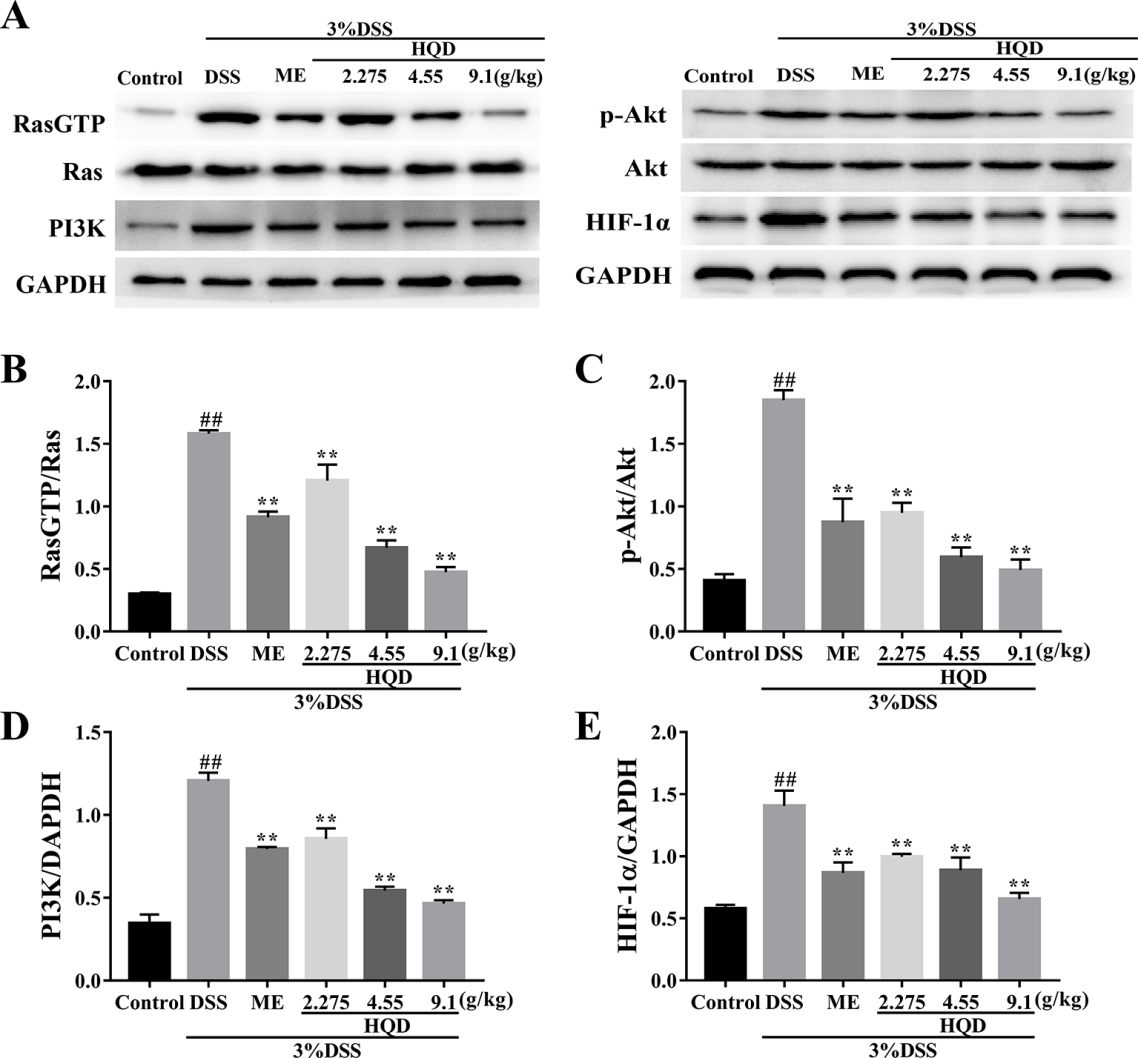
Effects of Huangqin decoction (HQD) on colonic expression levels of Ras-phosphoinositide-3-kinase (PI3K)-Akt-hypoxia inducible factor 1 alpha (HIF-1α) pathway proteins assessed by Western blot in dextran sulfate sodium (DSS)-induced colitis mice **(A)**. The protein levels of Ras **(B)**, Akt **(C)**, PI3K **(D)**, and HIF-1α **(E)** were determined by densitometry analyses. Data are presented as the means ± SEM (*n* = 3). ^##^ *p* < 0.01 vs. control group; **p < 0.01 *vs*. DSS group.

Ras is a key effector that activates downstream signaling cascades when phosphorylated (Tuncel and Kalkan, 2018). As shown in **Figure 6**, phosphorylation of Ras was significantly increased in the DSS group compared to that in the control group (*p* < 0.01). The expression of RasGTP was significantly lower in the response to HQD treatment than that in the DSS group (*p* < 0.01), which resulted in suppression of the PI3K/Akt pathway. In addition, the expression of PI3K and p-Akt were reduced in HQD treated mice compared to that in DSS treated mice (*p* < 0.01). Furthermore, the expression of HIF-1α was significantly higher in the DSS group compared to that in the control group, and treatment with HQD reversed this increase (*p* < 0.01).

**Figure 7:**
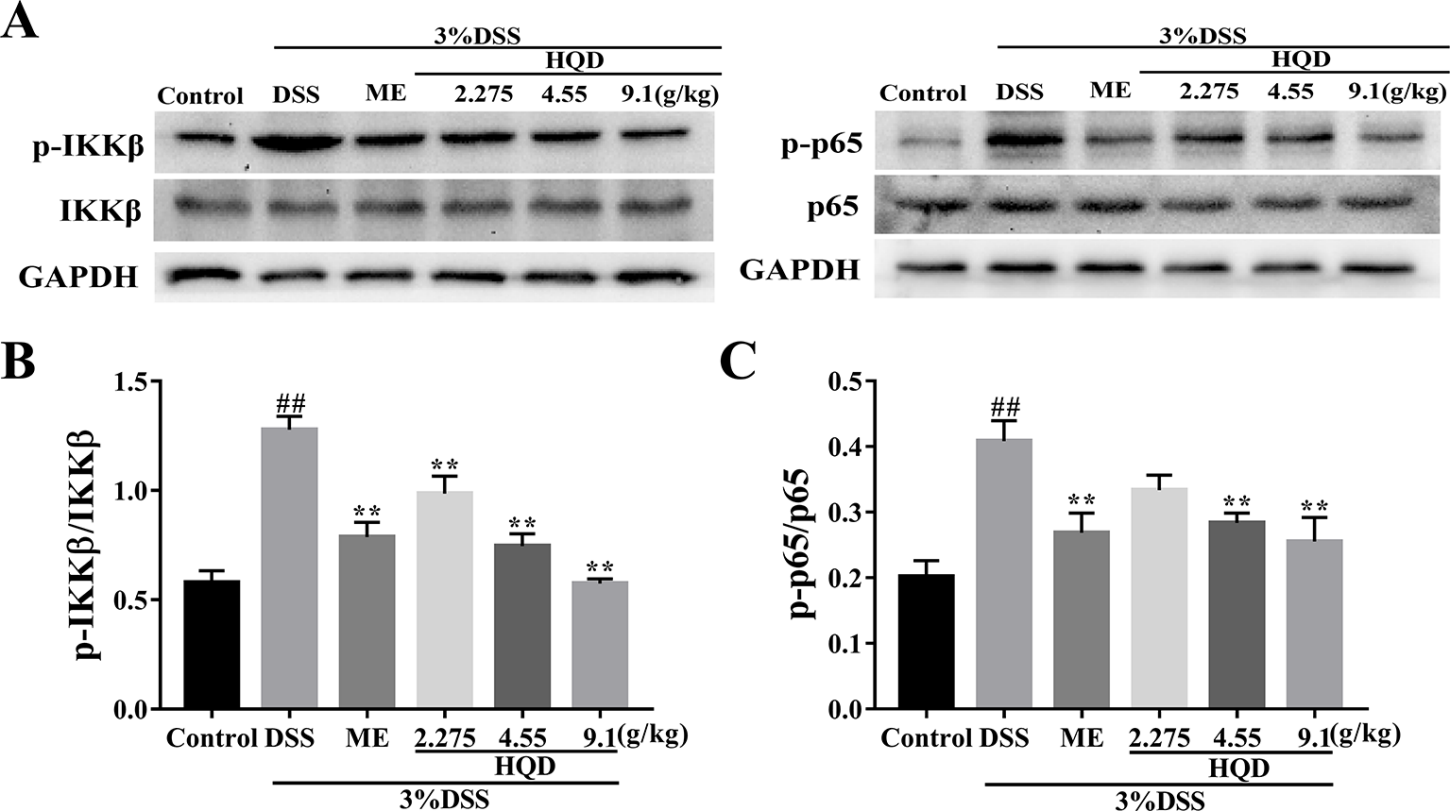
Effects of Huangqin decoction (HQD) on colonic expression levels of nuclear factor-kappa B (NF-κB) pathway proteins assessed by Western blot in dextran sulfate sodium (DSS)-induced colitis mice **(A)**. Densitometry analyses of Western blots were determined by quantifying the protein levels of total and phosphorylated inhibitor kappa B kinase β (IKKβ) **(B)** and p65 **(C)**. Data are presented as the means ± SEM (*n* = 3). ^##^ *p* < 0.01 vs. control group; **p < 0.01 *vs*. DSS group.

Phosphorylated Akt stimulates IKK activity, which results in activation of the NF-κB pathway (Dong et al., 2010). As shown in **Figure 7**, phosphorylation of NF-κB p65 and IKKβ was significantly increased in the DSS group compared to that in the control group (*p* < 0.01). Conversely, treatment with 4.55 and 9.1 g/kg of HQD inhibited the expression of NF-κB p-p65 and p-IKKβ in a dose-dependent manner (*p* < 0.01). Therefore, HQD alleviated DSS-induced colitis through downregulation of the Ras-PI3K-Akt-HIF-1α pathway, which resulted in inhibition of the NF-κB pathway.

### Huangqin Decoction Altered Overall Structural Modulation of Gut Microbiota

**Figure 8:**
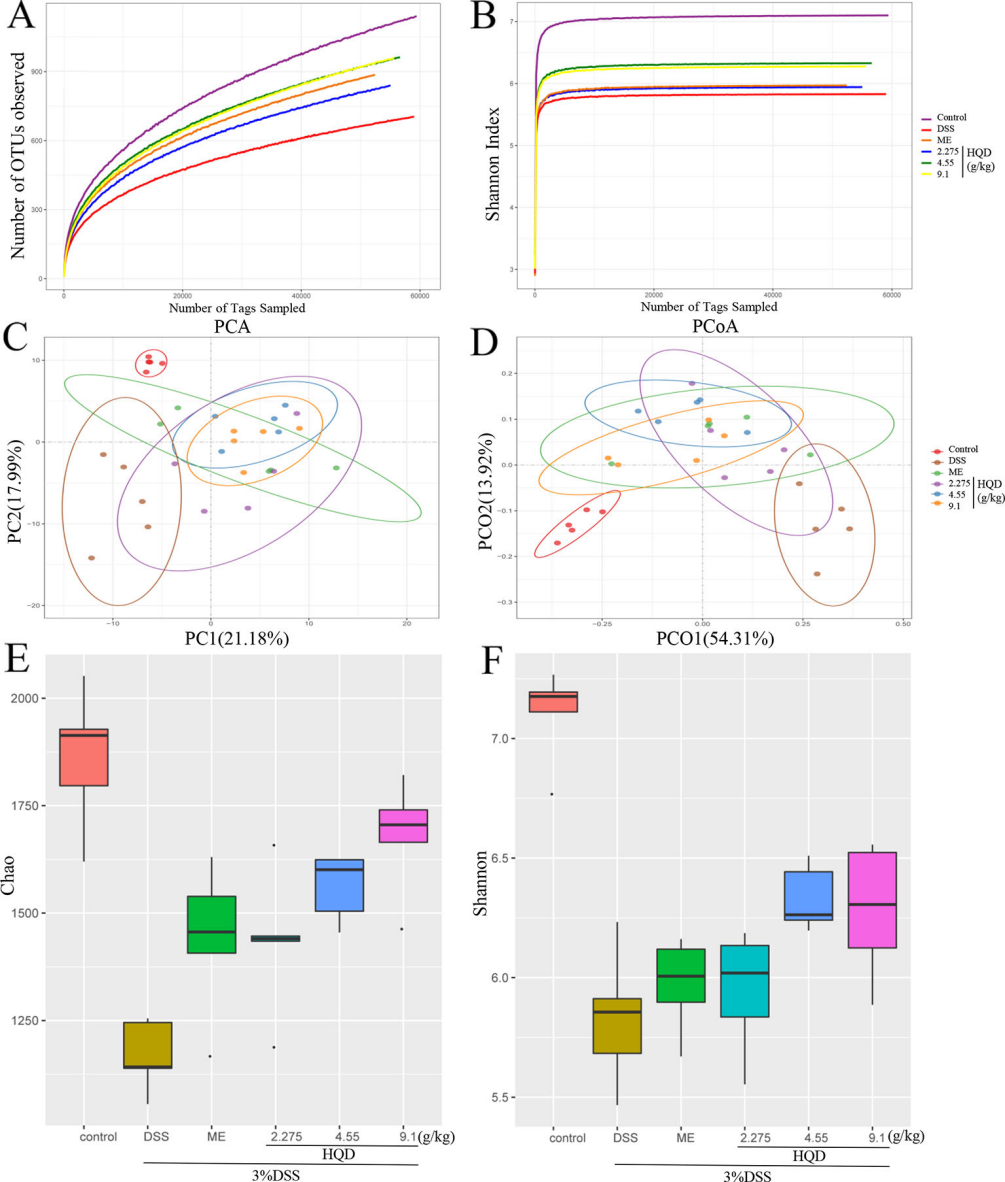
Effects of Huangqin decoction (HQD) on overall structural modulation of gut microbiota in dextran sulfate sodium (DSS)-induced colitis mice. **(A)** Rarefaction curve of operational taxonomic units (OTUs). **(B)** Shannon rarefaction curve of samples. **(C)** Multiple sample principal component analysis (PCA). **(D)** Multiple sample principal coordinate analysis (PCoA). **(E)** Chao and **(F)** Shannon of alpha diversity analysis (*n* = 5).

Structural changes in the gut microbiota have been reported in patients with UC and in DSS-induced colitis models (Ohkusa and Koido, 2015). As shown in **Figures 8A, B**, a plateaued rarefaction curve of OTUs indicated that the sequencing depth covered all the species in the samples. We showed that the DSS group had significantly less OTUs than the control group. The DSS-induced decrease in OTUs was reversed by HQD in a dose-dependent manner. Principal component analysis (PCA) and principal coordinate analysis (PCoA) showed similarity among samples, with similarity indicated by distance in the diagrams. Treatment with DSS altered the composition and structure of the gut microbiota according to PCA and PCoA. Treatment with HQD partially inhibited DSS-induced changes in the gut microbiota (**Figures 8C, D**). Alpha diversity analysis showed a clear decline in bacterial diversity in response to DSS treatment, and a clear increase in diversity in response to HQD treatment (**Figure 8E**).

### Huangqin Decoction Regulated Structural Segregation of the Gut Microbiota in Mice

**Figure 9:**
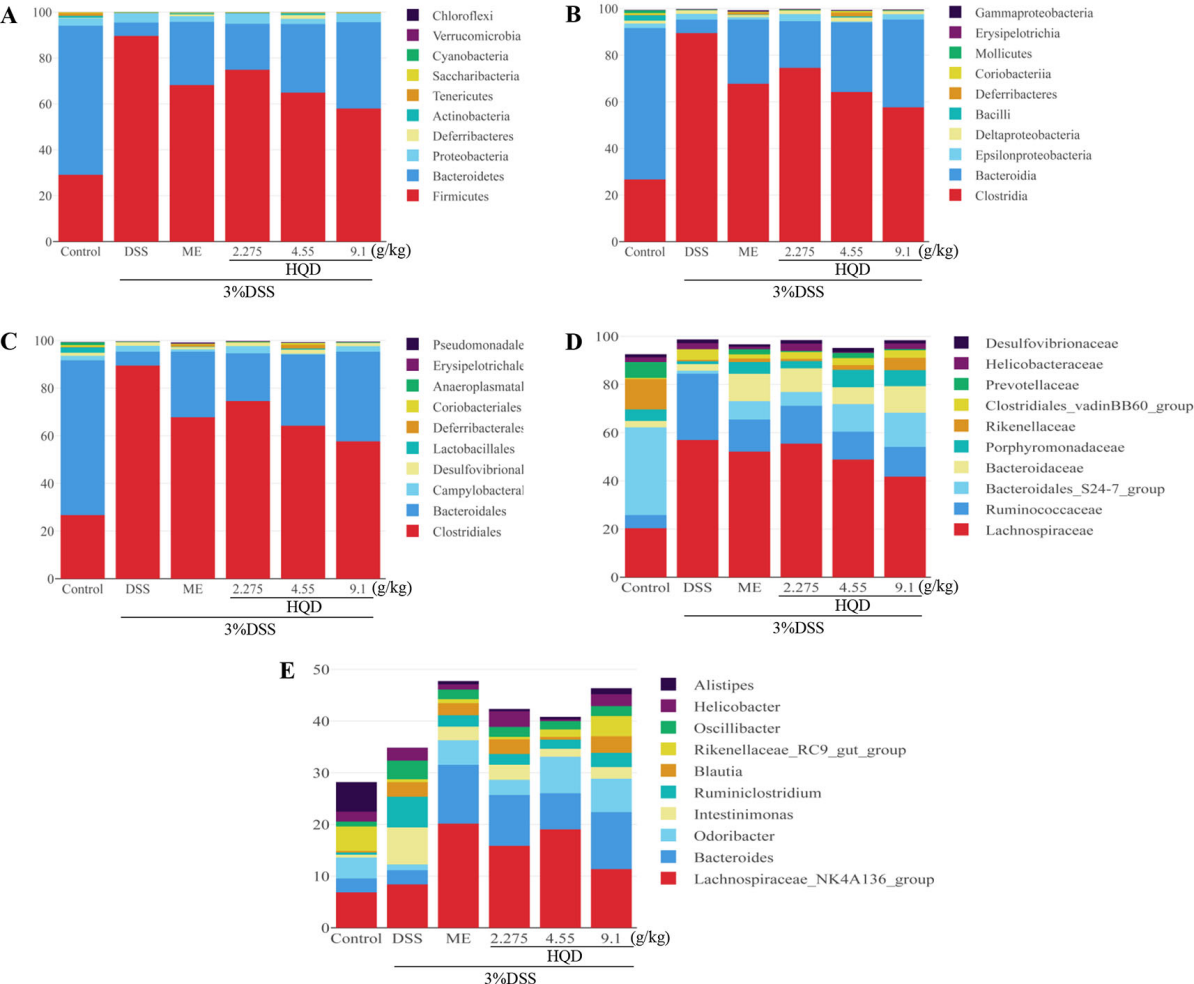
Effects of Huangqin decoction (HQD) on structural segregation of gut microbiota in dextran sulfate sodium (DSS)-induced colitis mice. **(A)** Phylum, **(B)** class, **(C)** order, **(D)** family, and **(E)** genus (*n* = 5).

The gut microbiota community structure was reported using histograms at the phylum, class, order, family, and genus levels (**Figure 9**). All samples contained abundant Bacteroidetes, Firmicutes, and Proteobacteria. Compared to the control group, DSS decreased the relative abundance of Bacteroidetes (*p* < 0.01) and increased the levels of Firmicutes (*p* < 0.01) and Proteobacteria. Treatment with HQD reduced these DSS-induced changes.

Fourteen classes including *Clostridia*, *Bacteroidia*, *Epsilonproteobacteria*, and *Deltaproteobacteria* were found in all samples (**Figure 9**). The results showed that the proportions of *Epsilonproteobacteria* and *Deltaproteobacteria* returned to control levels following HQD treatment. In addition, the relative abundance of *Bacilli* and *Coriobacteriia* drastically dropped in the DSS group, but increased significantly following treatment with HQD (*p* < 0.01).

Sequencing data identified 15 orders of gut microbes (**Figure 9**). *Lactobacillales*, *Deferribacterales*, and *Coriobacteriales* were expressed at high levels in the HQD group, but at low levels in the DSS group (all *p* < 0.01). Sequencing data identified 23 families of microbial flora, which is similar to the observations made at the phylum and class levels. As shown in **Figure 9D**, *Lachnospiraceae* and *Ruminococcaceae* were dominant communities in the DSS group, but were reduced by HQD treatment. Compared with the control group, *Bacteroidales_S24-7* and *Porphyromonadaceae* were observed at much lower levels in the DSS group (*p* < 0.01), and HQD protected against DSS-induced downregulation of these families.

**Figure 10:**
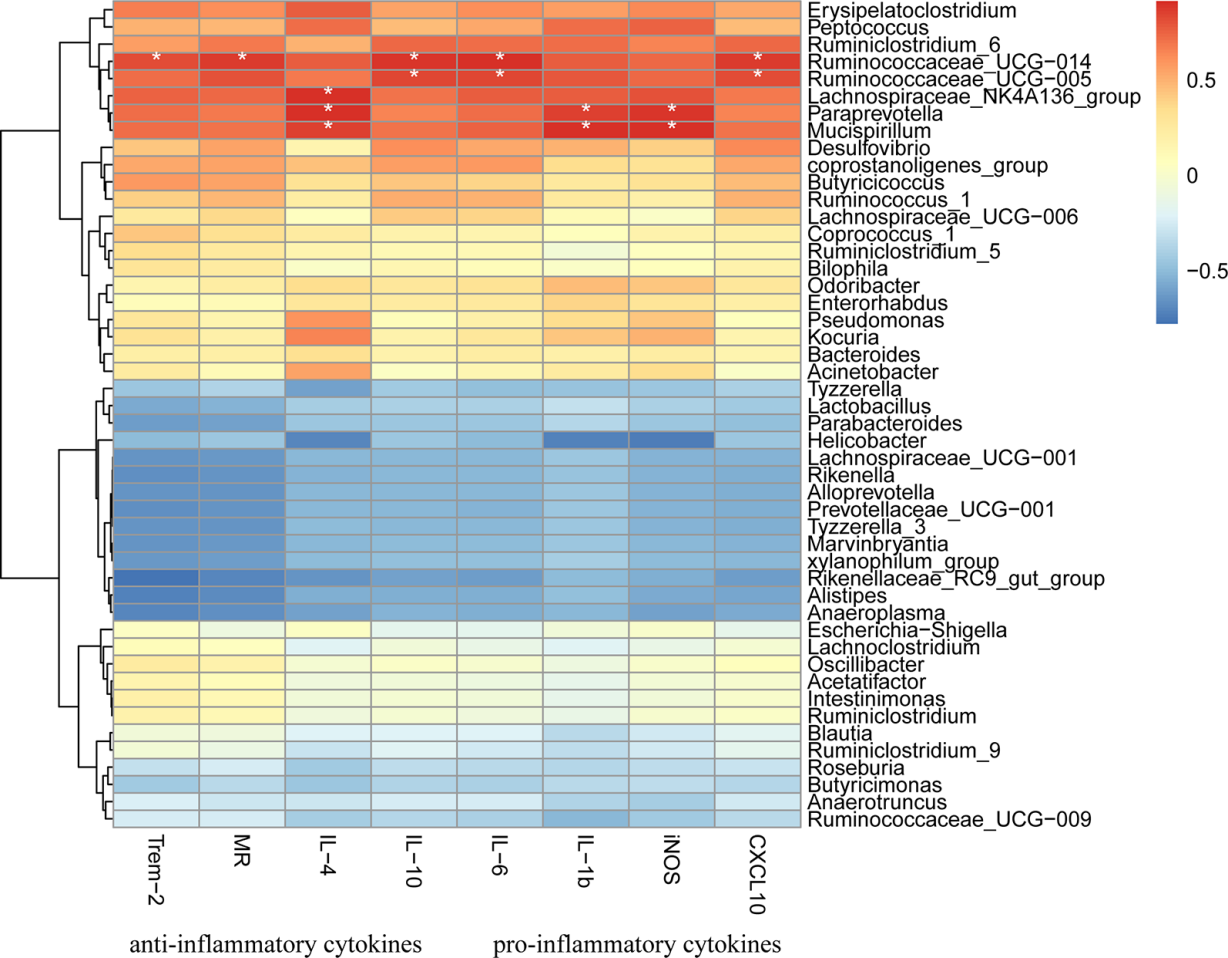
Heatmap presentation of Pearson correlation analysis between bacterial genera and inflammatory cytokines. Red color means positive correlation, blue color means negative correlation whereas white color means no correlation. * *p* < 0.05.

Finally, 47 genera were identified in all samples (**Figure 9**). The relative abundances of *Bacteroides*, including *Odoribacter* and *Alistipes*, were significantly down-regulated in response to DSS treatment (*p* < 0.01), and HQD treatment reversed these decreases. We used Pearson correlation analysis to evaluate associations between bacterial genera and inflammatory mediators. The results indicated that *Ruminococcaceae_UCG-014*, *Ruminococcaceae_UCG-005*, and *Lachnospiraceae_NK4A136*, which belong to Firmicutes phylum, were positively associated with inflammatory cytokines (**Figure 10**). In addition, *Paraprevotella* and *Mucispirillum* were more closely associated with pro-inflammatory cytokines than with anti-inflammatory cytokines.

## Discussion

In this study, we demonstrated that HQD could be used to treat DSS-induced colitis through regulation of the Ras-PI3K-Akt-HIF-1α pathway. We used a DSS-induced colitis model to study UC. This model is more representative of UC than trinitrobenzene sulfonic acid (TNBS)-induced colitis, and its symptoms more closely resemble clinical manifestations (Philip et al., 2010; Li et al., 2016). We used this model to identify the molecular mechanism of the HQD-mediated anti-inflammatory effects against DSS-induced colitis in mice.

Patients with UC are generally administered anti-inflammatory or immunosuppressive medicines (Ross and Cohen, 2004). However, we showed that HQD ameliorated DSS-induced colitis more effectively than ME. Body weight loss, DAI score, and colon shortening are often recognized as inflammatory markers (Feifei et al., 2015). In our study, treatment with HQD reduced these inflammatory indices in a dose-dependent manner. The results showed that HQD at a dose of 9.1 g/kg exerted significant protective effects against the inflammatory components of DSS-induced colitis.

The gut microbiota is comprised of many microorganisms such as bacteria, viruses, and fungi, and the gastrointestinal microbiome plays an important role in intestinal diseases such as IBD and CRC. Our results showed that DSS reduced the diversity of the gut microbiota, which was consistent with the results of previous studies (Hager and Ghannoum, 2017). Furthermore, DSS reduced the relative abundance of beneficial bacteria, and increased the abundance of pathogenic bacteria, compared with the control group. In contrast, HQD promoted recovery of the diversity of the gut microbiota, and regulated the populations of beneficial bacteria and pathogenic bacteria, as evidenced by higher levels of *Bacteroides*, *Lactobacillus*, *Prevotellaceae*, and *Blautia*, and lower levels of *Epsilonproteobacteria*, *Deltaproteobacteria*, and *Helicobacter*. *Ruminococcus* is regarded as a benign bacteria, and was inversely correlated with intestinal permeability in a previous study (Shang et al., 2016). In our study, the relative abundance of *Ruminococcus* in the DSS group was higher than that in the HQD group. We hypothesized that *Ruminococcus* may promote disease. These results suggested that HQD regulated the gut microbiota to maintain intestinal flora homeostasis in DSS-induced colitis.

Dysbiosis of gut microbiota signals to epithelial cells is associated with destruction of the intestinal barrier (Jackson and Theiss, 2019), which impairs barrier function and triggers inflammation (Ohkusa and Koido, 2015). Increased apoptosis in gut epithelial cells results in damage to the mucosal barrier (Antonio et al., 2003), which increases intestinal permeability and decreases MUC2 production. Goblet cells play a critical role in protection against DSS-induced colitis through secretion of a variety of mucus to strengthen the colonic barrier (Chen et al., 2018). Goblet cell depletion and epithelial barrier attenuation are common pathologies associated with UC. We evaluated colonic epithelial cells using H&E staining and goblet cells using PAS staining. Treatment with HQD significantly reversed DSS-induced histopathological changes in the epithelial barrier, including inflammatory infiltration, enterocyte loss, and disruption of crypt structures. Furthermore, treatment with HQD improved the intestinal barrier and increased goblet cell number. Infiltration of neutrophils plays a key role in pathogenesis of DSS-induced colitis (Nishitani et al., 2009), and increased MPO activity represents neutrophil accumulation. MPO activity was low in mice treated with HQD. Our results showed that HQD alleviated DSS-induced acute colitis by improving the intestinal epithelial cell barrier, preventing infiltration of inflammatory cells, and increasing goblet cell number.

LPS, the principal component of the cell wall of gram-negative bacteria, activates signaling pathways by binding to the TLR4 receptor, which results in activation of macrophages and release of immunomodulatory molecules (West et al., 2006). Activation of the TLR4 receptor results in activation of Ras, which signals through PI3K and Akt (Yoo et al., 2017). GPCR are activated by short-chain fatty acids (SCFA), which are metabolic products of bacteria (Sun et al., 2017), resulting in activation of Ras and PLC (Xie et al., 2016). Phospholipase C regulates activation of class I PI3K (Sabine et al., 2012). In our study, increased activation of TLR4 and GPCR in the DSS group resulted in a strong inflammatory response. Treatment with HQD mitigated this response (**Figure 4**). These results suggested that the anti-inflammatory effects of HQD against DSS-induced colitis might be mediating through TLR4 and GPCR.

Ras, the prototypical member of the Ras superfamily, plays an important role in regulation of immunity and inflammation (Johnson and Chen, 2012). Activated Ras activates the PI3K/Akt, Raf/MEK/ERK, and STAT pathways. The PI3K/Akt pathway enhances production of HIF-1α *via* activation of mammalian target of rapamycin (mTOR) (Land and Tee, 2007). Studies have shown that HIF-1α and NF-κB regulate the inflammatory microenvironment (Chen et al., 2015). In this study, we evaluated the anti-inflammatory effects of HQD against DSS-induced colitis, and showed that these effects occurred through the Ras-PI3K-Akt-HIF-1α pathway. The key proteins RasGTP, PI3K, p-Akt, and HIF-1α were highly expressed in the DSS group, and treatment with HQD decreased the expression of these proteins. In addition, HQD suppressed the NF-κB pathway by lowering the expression levels of p-IKKβ and NF-κB p-p65. These results showed that HQD exerted anti-inflammatory effects by suppressing the Ras-PI3K-Akt-HIF-1α and NF-κB pathways.

Macrophages undergo classical M1 activation or alternative M2 activation in response to different signals (Li et al., 2019). The M1 macrophage phenotypic markers iNOS and CXCL10 are associated with pro-inflammatory cytokines (IL-1β, IL-6), and the M2 macrophage phenotypic markers MR2 and Trem2 are associated with anti-inflammatory cytokines (IL-4, IL-10) (Huang et al., 2017). High levels of chemokines and pro-inflammatory cytokines, such as CXCL10 and IL-1β, mediate cellular infiltration, resulting in colonic tissue damage (Wen et al., 2014). In contrast, an abundance of anti-inflammatory cytokines results in tissue repair and resolution of inflammation (Luzina et al., 2012). We showed that HQD reduced the expression of the inflammatory mediators measured in this study, and upregulated the expression of anti-inflammatory cytokines and suppressed the expression of pro-inflammatory cytokines. We showed that HQD ameliorated DSS-induced colitis by producing an anti-inflammatory environment that promoted colon tissue repair.

*Ruminococcaceae*, *Lachnospiraceae*, *Paraprevotella*, and *Mucispirillum* were closely associated with inflammatory mediators, as determined using Pearson correlation analysis (**Figure 9**). These results suggested that inflammatory cytokines act upon intestinal flora. A previous study showed that IL-1β elevated the expression of Ras and HIF-1α by acting on the IL-1β-MyD88-Ras-NF-κB-HIF-1α axis (Kaluz and Meir, 2011). Therefore, the protective effects of HQD against DSS-induced colitis might be closely associated with suppression of inflammatory cytokine production, and the resultant effects on the immune microenvironment and the gut microbiota.

## Conclusion

Our study highlighted the anti-inflammatory effects of HQD in DSS-induced colitis. Treatment with HQD improved the gut microenvironment and exerted anti-inflammatory effects in mice with DSS-induced colitis. At the cellular level, HQD improved the intestinal epithelial cell barrier by preventing infiltration of inflammatory cells, and increasing goblet cell number. At the molecular level, HQD suppressed the Ras-PI3K-Akt-HIF-1α and NF-κB pathways, resulting in down-regulation of inflammatory cytokines. Our investigation provided experimental evidence that HQD may act as a potential candidate for treatment of UC.

## Data Availability Statement

The raw data generated for this article can be accessed from NCBI, using the accession number PRJNA588511.

## Ethics Statement

All animal study protocols were carried out in accordance with the approved guidelines from the Animal Experimentation Ethics Committee at Guangzhou University of Traditional Chinese Medicine (20181222002).

## Author Contributions

M-YL designed the study, collected and analyzed the data, and wrote the manuscript. XW, Y-HL, Y-XG, and Y-MZ contributed to data analyze. NX, S-HZ, C-LZ, and H-JL performed the experiment. Z-RS, X-BZ, and X-QH supervised the study. All authors reviewed and approved the manuscript.

## Funding

This work was supported by Provincial-level Major Scientific Research Projects in Regular Universities of Guangdong Province, China (No. 2017KZDXM017), Social Science and Technology Development Major Project of Dongguan City, China (No. 20185071201131614), Guangzhou University of Chinese Medicine Discipline Research Characteristic Training Project (No. XKP2019007), Special Project on the Integration of Industry, Education and Research of Guangdong Province (2014B090902002), Guangdong Provincial Key Laboratory of New Drug Development and Research of Chinese Medicine (2017B030314096).

## Conflict of Interest

The authors declare that the research was conducted in the absence of any commercial or financial relationships that could be construed as a potential conflict of interest.

## Abbreviations

CXCL10, CXC Chemokine Ligand 10; DSS, Dextran Sulfate Sodium; GPCR, G Protein-Coupled Receptors; HIF-1α, Hypoxia Inducible Factor 1 Alpha; HQD, Huangqin Decoction; Inos, Inducible Nitric Oxide Synthase; IL-1β, Interleukin-1β; IL-4, Interleukin-4; IL-6, Interleukin-6; IL-10, Interleukin-10; Ikkβ, Inhibitor Kappa B Kinase B; LPS, Lipopolysaccharide; ME, Mesalazine; MPO, Myeloperoxidase; MR, Mannose Receptor; NF-κb, Nuclear Factor-Kappa B; UC, Ulcerative Colitis; PI3K, Phosphoinositide-3-Kinase; TLR4, Toll-Like Receptor 4; Trem2, Triggering Receptor Expressed on Myeloid Cells 2.
