# Supplementary material for: Anti-Inflammatory Effects of Huangqin Decoction on Dextran Sulfate Sodium-Induced Ulcerative Colitis in Mice Through Regulation of the Gut Microbiota and Suppression of the Ras-PI3K-Akt-HIF-1α and NF-κB Pathways
Source: Front Pharmacol. 2020 Jan 20;10:1552. doi: 10.3389/fphar.2019.01552 (PMC6984456; doi:10.3389/fphar.2019.01552)
Supplement: Supplementary file 1 [file Table_1.pdf]

Supplemental Table 1: The water intake of mice in each group

Group	Water Intake (mL)						
	Day1	Day2	Day3	Day4	Day5	Day6	Day7
Control	69.1	69.7	70.0	66.8	68.6	69.1	68.9
DSS	69.7	69.0	69.1	63.0	64.4	63.8	62.4
DSS+ME	68.6	69.5	68.3	63.0	63.0	64.9	63.7
DSS+2.275g/kg HQD	69.3	69.7	68.3	65.5	64.8	65.0	64.5
DSS+4.55g/kg HQD	68.4	69.1	69.5	68.5	67.4	68.2	67.6
DSS+9.1g/kg HQD	68.8	69.3	67.8	68.6	67.0	68.9	68.6
